# Differentiating gallbladder cancer from benign gallbladder diseases using image-guided percutaneous gallbladder biopsy: A retrospective single-center study of 28 cases

**DOI:** 10.1097/MD.0000000000045487

**Published:** 2025-11-14

**Authors:** Min Jae Kim, Keun Soo Ahn, Tae-Seok Kim, Yong Hoon Kim, Koo Jeong Kang, Mu Sook Lee, Mijeong Kim, Byoungje Kim, Young Hwan Kim, Hye Won Lee

**Affiliations:** a Department of Surgery, Division of Hepatobiliary and Pancreatic Surgery, Keimyung University Dongsan Hospital, Daegu, Republic of Korea; b Department of Radiology, Keimyung University Dongsan Hospital, Daegu, Republic of Korea; c Department of Pathology, Keimyung University Dongsan Hospital, Daegu, Republic of Korea.

**Keywords:** gallbladder cancer, histologic diagnosis, percutaneous biopsy, transhepatic core needle biopsy, xanthogranulomatous cholecystitis

## Abstract

Differentiating gallbladder cancer (GBC) from benign inflammatory conditions such as xanthogranulomatous cholecystitis remains challenging because of overlapping imaging features. Misdiagnosis may lead to unnecessarily extensive resection in benign cases or inadequate oncological treatment in malignant cases. Preoperative histological confirmation can improve diagnostic accuracy and support appropriate surgical decision-making. We retrospectively analyzed 28 patients who underwent image-guided percutaneous transhepatic core needle biopsy for radiologically indeterminate GB lesions with hepatic infiltration. The biopsy findings were classified as malignant, suspicious, or benign. Surgical and pathological outcomes were reviewed, and diagnostic performance was assessed. Concordance between serum tumor markers and frozen sections was evaluated. Biopsies yielded adequate tissue in all cases (100%). Histological results were malignant in 3 patients (10.7%), suspicious in 1 (3.6%), and benign in 24 (85.7%). The final pathology confirmed GBC in 4 patients (14.3%) and benign disease in 24 (85.7%). One malignancy was missed on the preoperative biopsy but was detected intraoperatively. The sensitivity, specificity, and overall diagnostic accuracy were 75.0%, 100.0%, and 96.4%, respectively. Major complications were not observed. Tumor marker levels did not differ significantly between the groups. Frozen sections were concordant with the final diagnosis in 8 of 9 cases (88.9%). Image-guided percutaneous GB biopsy is a safe and effective diagnostic tool for differentiating GBC from benign mimickers. When combined with intraoperative frozen section analysis in selected patients, it allows for optimized surgical planning while minimizing the risk of over- or undertreatment.

## 1. Introduction

Gallbladder cancer (GBC) and severe inflammatory gallbladder diseases, particularly xanthogranulomatous cholecystitis (XGC), often exhibit similar imaging findings, making preoperative differentiation challenging.^[1,2]^ Both conditions can show diffuse gallbladder wall thickening and infiltration into the adjacent hepatic parenchyma, which are features commonly associated with malignancy.^[3,4]^

Misdiagnosis of benign inflammatory conditions such as chronic cholecystitis or XGC as GBC can lead to unnecessary major hepatic resections and lymphadenectomy, increasing surgical morbidity. Conversely, underestimating malignancy and treating GBC as a benign disease may result in insufficient resection, potentially compromising oncological outcomes. This is particularly relevant given that chronic cholecystitis and large polyps are recognized risk factors for malignancy and should be approached with caution during preoperative planning.^[5]^ Thus, accurate preoperative diagnosis is essential to avoid overtreatment of benign diseases and ensure appropriate surgical planning for malignancies. However, these entities are often difficult to distinguish preoperatively.

Endoscopic access to the GB is limited, and the risk of perforation makes biopsy challenging. Endoscopic retrograde cholangiopancreatography-based sampling methods, such as bile cytology and brushing, have shown low sensitivity (<50%) for diagnosing GBC.^[6]^ Percutaneous fine-needle aspiration (FNA) has been reported since the 1990s; however, concerns regarding bile leakage, peritonitis, and tumor seeding have limited its widespread use. Furthermore, cytology alone often fails to provide a definitive histological distinction between cancer and inflammation.^[7]^

Traditionally, intraoperative frozen section analysis has been used to assess malignancy during surgery, allowing for intraoperative adjustment of the extent of resection.^[8]^ However, this approach requires surgery without a definitive preoperative diagnosis, which may lead to suboptimal planning. Recently, endoscopic ultrasound-guided fine-needle aspiration or biopsy (EUS-FNA/FNB) has emerged as a promising tool for the preoperative diagnosis of GB lesions and regional lymphadenopathy.^[9–13]^ Previous studies have suggested that obtaining sufficient core tissue, especially from areas infiltrating the liver, may improve diagnostic yield.^[10,11]^ Despite these advances, EUS-FNA/FNB requires specialized endoscopic equipment and trained personnel, which limits its availability in general practice.

In contrast, image-guided percutaneous needle biopsy is a widely available and minimally invasive method that is routinely used for abdominal organ sampling.^[14]^ When performed using a transhepatic approach and core biopsy needle, it can provide sufficient tissue for histological evaluation while reducing the risk of bile leakage by confining any potential leakage to the liver parenchyma.^[15]^

In this study, we retrospectively analyzed 28 cases in which image-guided percutaneous biopsy was performed preoperatively in patients with GB lesions showing hepatic infiltration and a radiologic suspicion of GBC. We aimed to evaluate the diagnostic accuracy and clinical value of this approach for distinguishing GBC from benign GB diseases, including chronic cholecystitis and XGC.

## 2. Methods

### 2.1. Study population

We analyzed data from 28 patients who underwent percutaneous transhepatic biopsy for suspected GBC at a single tertiary center between January 2008 and December 2023. All patients had GB lesions that showed marked wall thickening or mass-forming features on imaging, accompanied by hepatic parenchymal infiltration, which raised concerns about malignancy. These radiological findings were considered indeterminate, with differential diagnoses of GBC, XGC, and severe chronic cholecystitis. Radiology reports variably described the lesions as “suggestive of malignancy,” “cannot exclude malignancy,” or “possibly benign.” This was a retrospective case series without a control group.

The study was conducted in accordance with the Declaration of Helsinki and approved by the Institutional Review Board of Keimyung University Dongsan Hospital (IRB No. 2025-07-024). The requirement for written informed consent was waived due to the use of anonymized clinical data.

### 2.2. Biopsy procedure

All percutaneous biopsies were performed by board-certified interventional radiologists under ultrasonography or computed tomography (CT) guidance. In most cases, the transhepatic route was chosen to access the lesion through the hepatic parenchyma, particularly when the GB lesion was adjacent to or had infiltrated the liver. This approach was intended to minimize bile leakage into the peritoneal cavity by allowing any leakage to be confined to the liver tissue.

Under local anesthesia, core needle biopsy was performed using an 18-gauge or 20-gauge needle. A total of up to 12 core tissue samples were obtained from the central part of the lesion or the area of hepatic infiltration. In some cases, an additional 22-gauge FNA was performed for cytologic smears, but the final diagnosis was based on the histologic results from the core biopsy.

All patients were monitored during and after the procedure for signs of immediate complications, such as bleeding or bile leakage. Clinical symptoms, including fever and abdominal pain, were followed up for 2 to 3 days after the procedure to identify any delayed complications. All the patients underwent surgical resection, which was performed when intraoperative frozen section analysis was feasible.

### 2.3. Data collection and outcome measures

Clinical parameters, including age, sex, imaging findings, serum levels of tumor markers (carbohydrate 19-9 and carcinoembryonic antigens), biopsy results, type of surgery performed, and final pathological diagnosis, were reviewed from electronic medical records. The pathologists classified the biopsy results as “malignant,” “suspicious for malignancy (atypical cells),” or “benign.”

The diagnostic accuracy of preoperative biopsy was assessed by comparing the biopsy results with the final surgical pathology (gold standard). The sensitivity, specificity, and overall accuracy were calculated. Cases categorized as “suspicious for malignancy” were considered positive for the sensitivity analysis based on their high clinical relevance and malignant potential. The presence or absence of procedure-related complications was also recorded and described.

### 2.4. Statistical analysis

The diagnostic performance of percutaneous biopsy was assessed by comparing the preoperative biopsy results with the final surgical pathology, which served as the gold standard. The sensitivity, specificity, and overall accuracy were calculated using standard 2 × 2 contingency tables. The biopsy results were classified as “disease,” malignant,” or “suspicious for malignancy” and were considered positive for the purpose of sensitivity analysis. Patients with final benign pathology and biopsy findings were considered true negatives.

Continuous variables such as serum carcinoembryonic antigen and carbohydrate antigen 19-9 levels were compared between the benign and malignant groups using the Mann–Whitney *U* test because of non-normal distribution. Statistical significance was set at *P* < .05. Statistical analyses were performed using SPSS Statistics for Windows version 28 (IBM Corp., Armonk).

## 3. Results

### 3.1. Patient characteristics

A total of 28 patients were included in the present study, comprising 13 males (46.4%) and 15 females (53.6%) with a median age of 70 years (range, 38–89 years) (Table 1). The most common presenting symptoms were right upper quadrant abdominal pain, fever, and jaundice; however, no specific clinical features could reliably distinguish between malignant and benign diseases.

**Table 1:** Summary of patient characteristics, biopsy results, and final diagnosis (N = 28).

Variable	Total (N = 28)
Age (yr), median (range)	70 (38–89)
Sex, n (%)	
Male	13 (46.4%)
Female	15 (53.6%)
Radiologic impression	
Suggestive of malignancy	7 (25.0%)
Indeterminate	20 (71.4%)
Likely benign	1 (3.6%)
Biopsy result	
Malignant	3 (10.7%)
Suspicious (favor malignant)	1 (3.6%)
Benign	24 (85.7%)
Surgical approach	
Extended cholecystectomy	4 (14.3%)
Open cholecystectomy	12 (42.9%)
Converted lap-to-open	12 (42.9%)
Final pathology diagnosis	
Gallbladder cancer	4 (14.3%)
Benign disease	24 (85.7%)
Complications	
Serious complication	0 (0%)
Minor pain	1 (3.6%)

* Values are presented as number (%) unless otherwise specified. “Suspicious” refers to biopsy findings favoring malignancy without definitive features.

**Figure 1.**
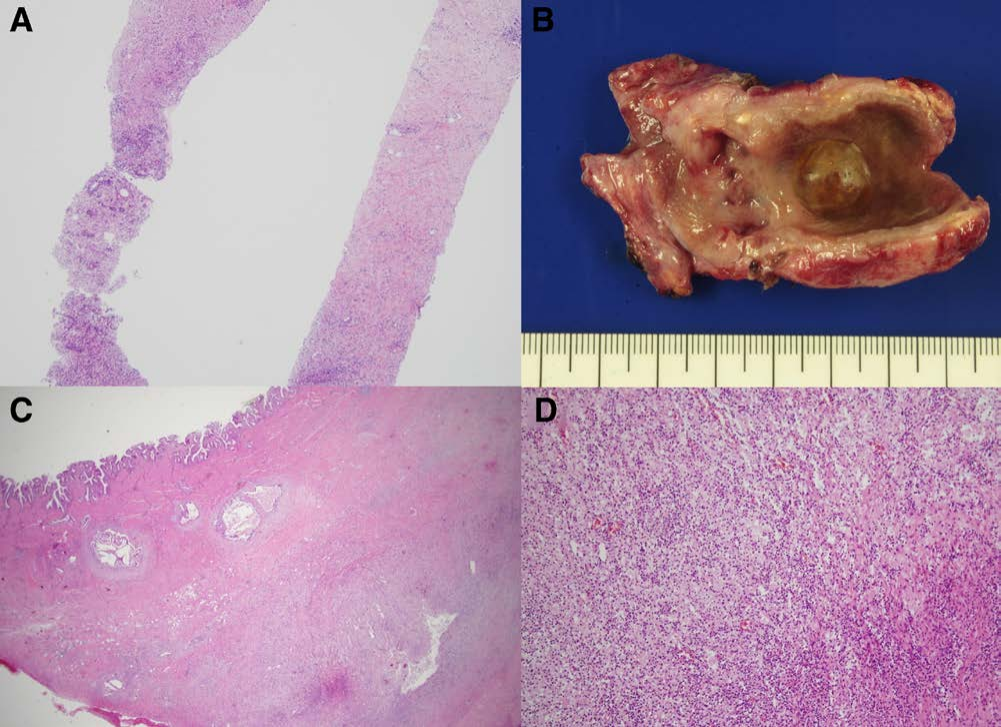
Axial and coronal computed tomography (CT) images of a gallbladder lesion. Marked gallbladder wall thickening with intramural low-density areas was noted along with obscured fat planes between the gallbladder and transverse colon, raising suspicion of complicated cholecystitis or malignancy. These findings prompted an image-guided percutaneous biopsy.

Radiologic interpretation classified 7 patients (25.0%) as “suspicious for malignancy,” 20 (71.4%) as “indeterminate,” and 1 (3.6%) as “likely benign.” Representative CT images showing GB wall thickening and pericholecystic changes are presented in Figure 1. Owing to these diagnostic uncertainties, all patients underwent image-guided percutaneous GB biopsy prior to surgery. Biopsy results revealed 24 benign cases (85.7%), 3 malignant cases (10.7%), and 1 suspected malignancy (3.6%).

Surgical approaches included open cholecystectomy in 12 patients (42.9%), laparoscopic-to-open conversion in another 12 (42.9%), and extended cholecystectomy in 4 (14.3%). Final pathological evaluation confirmed GBC in 4 patients (14.3%) and benign disease in the remaining 24 (85.7%).

Postoperative outcomes were favorable, with only 1 patient (3.6%) experiencing minor pain, and no serious complications or perioperative mortality. These findings highlight the safety and diagnostic value of combining preoperative biopsy with surgical intervention for the management of suspicious gallbladder lesions.

### 3.2. Biopsy findings and surgical outcomes

**Figure 2.**
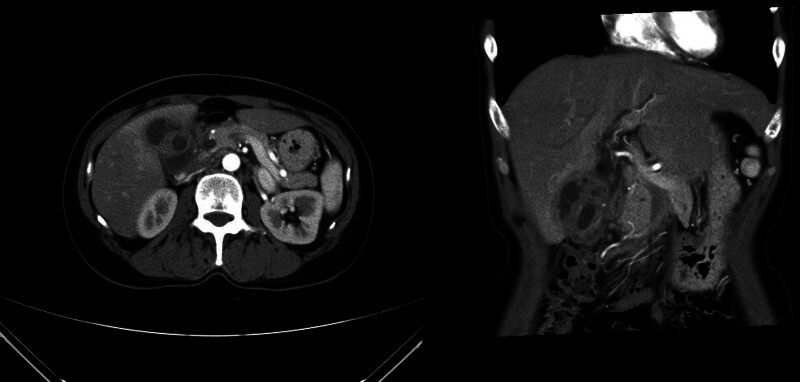
Axial computed tomography (CT) images showing percutaneous gallbladder biopsy using an 18-gauge core needle under imaging guidance. CT-guided transabdominal percutaneous biopsy of the gallbladder was performed to differentiate between complicated cholecystitis, xanthogranulomatous cholecystitis, and gallbladder cancer. Two core specimens were obtained using an 18-gauge needle without any procedure-related complications.

All 28 patients successfully underwent image-guided percutaneous core needle biopsy with a tissue acquisition rate of 100%. The percutaneous biopsy procedure is illustrated in Figure 2. Histopathological results were reported as “malignant” in 3 cases (10.7%), “suspicious for malignancy” in 1 (3.6%), and “benign” in 24 (85.7%).

All the patients underwent surgical resection. Four patients with biopsy results suggestive of malignancy or suspicion underwent extended cholecystectomy with hepatic wedge resection and regional lymphadenectomy. Among the 24 patients with benign biopsy results, 12 underwent an attempted laparoscopic cholecystectomy, which required conversion to open surgery in all cases because of severe inflammation. The remaining 12 patients underwent upfront open cholecystectomy based on preoperative imaging findings.

**Figure 3.**
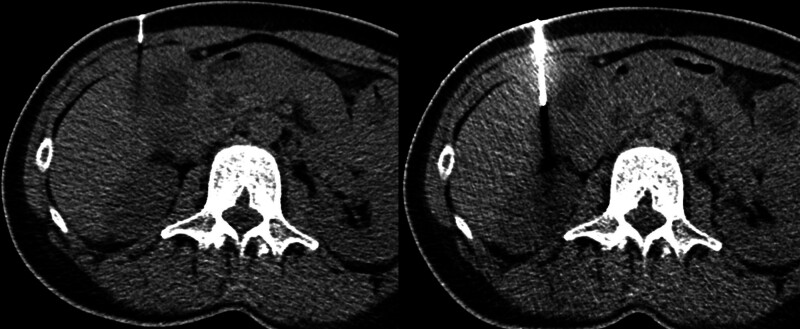
Histopathological and gross findings of gallbladder specimens. (A, C) Hematoxylin and eosin (H&E) staining (×40). Gallbladder biopsy shows diffuse infiltration of mixed inflammatory cells into the fibrous stroma without evidence of malignancy, suggestive of cholecystitis. (B) Gross examination reveals marked thickening of the gallbladder wall with an irregular mucosal surface covered by necrotic material. (D) H&E staining (×100). The surgical specimen demonstrates partial denudation of the mucosal epithelial lining without dysplasia. Extensive infiltration of foamy histiocytes and mononuclear cells is seen within a collagenous and fibroblastic background.

Histopathologic and gross findings of representative benign specimens, including XGC, are shown in Figure 3. Final pathology confirmed gallbladder adenocarcinoma in 4 patients (14.3%) and benign inflammatory conditions such as chronic cholecystitis, XGC, or IgG4-related cholecystitis in 24 (85.7%).

### 3.3. Diagnostic performance of image-guided biopsy

Preoperative biopsy findings were concordant with the final surgical pathology findings in 27 of the 28 patients (96.4%). One case of malignancy was misclassified as benign on the preoperative biopsy. Accordingly, the sensitivity, specificity, and overall diagnostic accuracy of percutaneous core needle biopsy for differentiating malignant from benign GB lesions were 75.0%, 100.0%, and 96.4%, respectively.

### 3.4. Tumor marker levels and diagnostic concordance

Subgroup analysis was performed to compare the malignant (n = 4) and benign (n = 24) groups (Table 2). The median age was slightly higher in the malignant group (72 years, range: 68–89 years) than in the benign group (69 years, range: 38–83 years); however, this difference was not statistically significant (*P* = .413).

**Table 2:** Comparison between malignant and benign groups.

Variable	Malignant (N = 4)	Benign (N = 24)	*P*-value
Age (yr), median (range)	72 (68–89)	69 (38–83)	.413
CEA, median (ng/mL)	1.22 (0.91–2.49)	1.61 (0.50–4.35)	.786
CA 19-9, median (U/mL)	26.71 (10.2–108.6)	31.03 (3.4–372.0)	.25
Frozen section performed, n (%)	3 (75.0%)	11 (45.8%)	–
Frozen section-final concordance, n (%)	3/3 (100%)	6/6 (83.3%)	–
Preoperative biopsy-final concordance, n (%)	3/4 (75.0%)	24/24 (100%)	–

CA 19-9 = carbohydrate antigen 19-9, CEA = carcinoembryonic antigen.

* Values are presented as median (range) or number (%).

Tumor marker levels did not differ significantly between the 2 groups. The median serum carcinoembryonic antigen level was 1.22 ng/mL (range 0.91–2.49) in the malignant group and 1.61 ng/mL (range 0.50–4.35) in the benign group (*P* = .786). Similarly, the median carbohydrate antigen 19-9 level was 26.71 U/mL in the malignant group and 31.03 U/mL in the benign group (*P* = .250)

Intraoperative frozen section biopsy was performed in 14 patients (50.0%), including 3 of 4 malignant cases (75.0%) and 11 of 24 benign cases (45.8%). Among these, the frozen section results were concordant with the final pathology in all malignant cases (3/3, 100%) and 5 of 6 benign cases (83.3%).

Preoperative biopsy results demonstrated high concordance with the final histopathology; 3 out of 4 malignant cases (75.0%) and all benign cases (24/24, 100%) were accurately classified prior to surgery.

## 4. Discussion

This study demonstrated that image-guided percutaneous GB biopsy offers high diagnostic accuracy and is a valuable tool for preoperative planning in patients with radiologically indeterminate GB lesions. In our retrospective analysis of 28 patients, the concordance between preoperative biopsy and final surgical pathology was 96.4%, with a sensitivity of 75.0% and specificity of 100.0%. Notably, percutaneous biopsy enabled an accurate diagnosis without complications in nearly all patients, allowing the extent of resection to be tailored appropriately in malignant cases and avoiding unnecessary major surgery in benign conditions.

In only one case, malignancy was missed on preoperative biopsy but was correctly identified by intraoperative frozen section, allowing the patient to undergo definitive oncologic surgery. These findings suggest that percutaneous biopsy can complement or potentially replace intraoperative frozen sections as a noninvasive diagnostic method in selected situations. When intraoperative frozen sections are selectively added to cases in which the biopsy result is benign or inconclusive, the likelihood of missing a malignancy may be further reduced.

Radiological differentiation between GBC and benign inflammatory conditions such as XGC remains inherently limited. Features, including GB wall thickening, pericholecystic fat stranding, and hepatic invasion, are commonly seen in both diseases, contributing to diagnostic ambiguity, even with advanced imaging modalities, such as contrast-enhanced CT and magnetic resonance imaging.^[1–4,16]^ Misinterpretation can lead to overtreatment with unnecessary extended resection in benign cases or, conversely, insufficient oncological surgery if the malignancy is underestimated.^[5]^

Historically, intraoperative frozen sections have been used to confirm malignancies during surgery; however, their use is limited. It requires partial tissue resection during surgery and carries risks, such as bleeding, GB perforation, and tumor seeding.^[8]^ Furthermore, frozen sections are not feasible in all settings and may not provide sufficient time for preoperative surgical planning. In our cohort, frozen sections were only selectively used, and although their concordance with the final pathology was high (88.9%), they cannot be universally relied upon.

In contrast, a preoperative percutaneous biopsy allows for rational surgical planning. In the current study, patients with benign biopsy findings underwent laparoscopic cholecystectomy when appropriate, whereas those with confirmed or suspected malignancies received extended resections from the outset. Notably, several patients with radiologic findings strongly suggestive of malignancy were able to avoid unnecessary liver resection or lymphadenectomy because of benign biopsy results. These findings support the routine use of preoperative percutaneous biopsies in patients with suspected GBC. When malignancy is confirmed, extended resection can be performed confidently. In cases with benign or indeterminate results, the selective use of intraoperative frozen sections may serve as an additional safeguard. This combined strategy may reduce both overtreatment and undertreatment, thereby enhancing the safety and precision of surgical management.

In addition to diagnostic precision, preoperative tissue confirmation can facilitate personalized treatment planning. A previous study has shown that conditional survival improves over time in patients with GBC, particularly in those who survive the early postoperative period.^[17]^ Thus, an accurate histological diagnosis may guide both the surgical extent and postoperative surveillance strategy according to individual risk profiles.

The diagnostic performance of percutaneous biopsy in our study was comparable to or exceeded the results reported for EUS-FNA/FNB. Prior studies have reported sensitivities of 93.3% to 97% and diagnostic accuracies of 80% to 97%.^[10,13]^ Although direct comparison is limited owing to the sample size and methodology, our findings support percutaneous biopsy as a practical and accurate alternative when adequate core tissue can be obtained. In our protocol, 18-to 20-gauge core needles enabled the histological evaluation of infiltrative glandular patterns, fibrosis, and other features required to differentiate between GBC and XGC.^[12,13]^ By targeting the hepatic-infiltrating portions of the lesion via a transhepatic route, we optimized the diagnostic yield while minimizing the risk of bile leakage or tract seeding.^[14]^

Our findings are consistent with those in the broader literature on procedural safety. GB interventions carry the risk of biliary peritonitis, particularly when bile leakage occurs in the peritoneal cavity.^[18]^ These risks can be mitigated through careful route planning and experienced technique.^[19]^ In our study, no major complications, such as tract seeding or bile peritonitis, were observed.

Notably, our results are aligned with current international recommendations. The 2023 European Society for Medical Oncology Clinical Practice Guidelines recommend the use of image-guided core needle biopsy in patients with suspected biliary tract malignancy, favoring histologic sampling over cytologic techniques owing to its higher yield and suitability for ancillary testing.^[19,20]^ The guidelines also acknowledge the low but manageable risk of tumor seeding and emphasize the importance of multidisciplinary decision-making in determining biopsy indications and routes. The use of the transhepatic approach in all cases was consistent with the safety guidelines.

These guideline-concordant findings reinforce the role of percutaneous biopsy not only as a diagnostic tool but also as an evidence-based and internationally endorsed component of preoperative planning for patients with suspected GBC. Early histological confirmation is particularly valuable because GBC frequently arises in the setting of chronic inflammation, including conditions such as cholelithiasis and chronic cholecystitis.^[5]^ Notably, early-stage GBC is associated with significantly better survival than advanced disease, underscoring the need for timely diagnosis and individualized treatment strategies based on tissue confirmation.

This study has several limitations. First, this was a retrospective, single-center analysis with a small sample size, which limits the generalizability of our findings and may preclude accurate estimation of rare complications. Second, the study focused exclusively on lesions with hepatic infiltration, and the findings may not be applicable to non-infiltrative GB lesions. Third, the absence of a direct comparison group, such as patients assessed with EUS-FNA or intraoperative frozen sections alone, limits the ability to compare diagnostic performance between modalities. Finally, surgical planning may have been influenced by clinical and radiologic judgments beyond the biopsy results, introducing potential confounders. Further prospective multicenter studies with larger cohorts are required to validate and extend our findings.

## 5. Conclusions

Image-guided percutaneous GB biopsy is a safe and effective preoperative diagnostic modality for evaluating suspected GB malignancies. It supports surgical decision-making by minimizing unnecessary extensive resections in benign conditions while ensuring appropriate oncologic treatment in malignant cases. When combined with intraoperative frozen section analysis in selected patients, this approach offers a reliable and balanced strategy to optimize outcomes in the management of complex GB pathology.

## Author contributions

**Conceptualization:** Min Jae Kim, Koo Jeong Kang.

**Data curation:** Min Jae Kim, Tae-Seok Kim, Yong Hoon Kim, Koo Jeong Kang, Mu Sook LEE, Mijeong Kim, Byoungje Kim, Young Hwan Kim, Hye won Lee.

**Formal analysis:** Hye won Lee.

**Investigation:** Min Jae Kim, Tae-Seok Kim.

**Methodology:** Min Jae Kim.

**Project administration:** Keun Soo Ahn.

**Supervision:** Keun Soo Ahn.

**Validation:** Keun Soo Ahn.

**Writing – original draft:** Min Jae Kim.

**Writing – review & editing:** Keun Soo Ahn.
